# Reprogramming the fate of human glioma cells to impede brain tumor development

**DOI:** 10.1038/cddis.2014.425

**Published:** 2014-10-16

**Authors:** Z Su, T Zang, M-L Liu, L-L Wang, W Niu, C-L Zhang

**Affiliations:** 1 Department of Molecular Biology, University of Texas Southwestern Medical Center, 6000 Harry Hines Boulevard, Dallas, TX 75390-9148, USA; 2 Institute of Neuroscience and Key Laboratory of Molecular Neurobiology of Ministry of Education, Neuroscience Research Center of Changzheng Hospital, Second Military Medical University, 800 Xiangyin Road, Shanghai 200433, China

## Abstract

Malignant gliomas, the most common solid tumors in the central nervous system, are essentially incurable due to their rapid growth and very invasive nature. One potential approach to eradicating glioma cells is to force these cells to undergo terminal differentiation and, in the process, to irreversible postmitotic arrest. Here, we show that neurogenin 2 (NGN2, also known as NEUROG2) synergizes with sex-determining region Y-box 11 (SOX11) to very efficiently convert human glioma cells to terminally differentiated neuron-like cells in both cell culture and adult mouse brains. These cells exhibit neuronal morphology, marker expression, and electrophysiological properties. The conversion process is accompanied by cell cycle exit, which dramatically inhibits glioma cell proliferation and tumor development after orthotopic transplantation. Most importantly, intracranial injection of NGN2- and SOX11-expressing virus into the tumor mass also curtails glioma growth and significantly improves survival of tumor-bearing mice. Taken together, this study shows a simple and highly efficient strategy for reprogramming malignant glioma cells into postmitotic cells, which might be a promising therapeutic approach for brain tumors.

Approximately 30% of primary tumors that develop in the central nervous system have features of transformed glial cells, such as astrocytes and oligodendrocytes, and are therefore defined as gliomas.^1^ They account for 80% of malignant brain tumors and are one of the most devastating forms of human cancer.^2^ Glioblastoma (astrocytoma WHO grade IV) is the most common primary glioma in the adult brain and is essentially incurable. Patients with glioblastoma only have a median survival time of 15 months.^3^ Despite therapeutic improvements through combined neurosurgery, chemotherapy, and radiotherapy, current treatment modalities are still unable to significantly prolong patients' survival. Therefore, the treatment of glioma continues to be a major challenge for neuro-oncologists.

The deadly nature of malignant glioma originates from its exponential growth and invasive behavior. One potential approach to blocking tumor growth and invasion is to induce them to become terminally differentiated cells. Indeed, previous studies demonstrate that glioma cells can be induced to undergo glial differentiation by the microRNA (miR)-146a,^4^ miR-34a,^5^ activation of the bone morphogenic protein signaling,^6,7^ all-trans retinoic acid,^8^ or small molecules targeting mutant isocitrate dehydrogenase 1 or inhibitors of apoptosis proteins.^9,10^ The miR-124 and miR-137 are also able to induce glioma cells to adopt a neuron-like fate.^11^ As cell fates are directly controlled by fate-determining transcriptional factors, a dominant way to reprogram a cell's fate is to change the composition of these factors. This is exemplified by the derivation of induced pluripotent stem cells from somatic cells by the overexpression of *Sox2*, *Oct4*, *Klf4*, and *c-Myc*.^12^ Accordingly, non-neuronal somatic cells, including glial cells, can be directly converted into neurons by neurogenic transcription factors.^12, 13, 14, 15, 16, 17, 18, 19, 20, 21, 22^ These results raised a potential that the behavior of malignant glioma cells may be similarly reprogrammed. Supporting this hypothesis is a recent report demonstrating that overexpression of three neurogenic transcription factors (achaete-scute complex homolog 1 (ASCL1), BRN2 and NGN2) in cultured human glioma cells is able to convert 20–40% of them into *β*III-tubulin (TUBB3, also known as TUJ1)-positive neuron-like cells.^23^ To effectively inhibit tumor growth, however, much higher conversion efficiency will be essential.

Previously, we identified that NGN2 and sex-determining region Y-box 11 (SOX11) can cooperatively reprogram postnatal or adult human skin fibroblasts to neurons with high efficiency (>90%). The reprogramming process is direct; it does not pass through a proliferative progenitor state.^24^ NGN2, a basic helix-loop-helix transcription factor, controls commitment of neural progenitors to neuronal fate during development.^25,26^ Ectopic expression of NGN2 alone is sufficient to convert early postnatal glial cells into neurons^13,27^ and induce proliferation arrest and massive death of CD133^+^ glioblastoma stem cells.^28^ Similarly, SOX11 is another essential regulator of neuronal fate that further promotes differentiation and survival.^29, 30, 31^ SOX11 was reported to prevent tumorigenesis of glioma-initiating cells by blocking the expression of oncogenic *Plagl1*.^32^ SOX11 expression serves as an indicator of favorable prognosis for patients affected by glioblastoma.^33^ Here we examined whether the fate of glioma cells could be reprogrammed by forced expression of NGN2 and SOX11. Our results show that these two factors synergistically and efficiently convert human malignant glioma cells into terminally differentiated neuron-like cells. This conversion process leads to significant inhibition of tumor growth in a mouse orthotopic transplantation model.

## Results

### Highly efficient conversion of human malignant glioma cells to neuron-like cells

**Figure 1:**
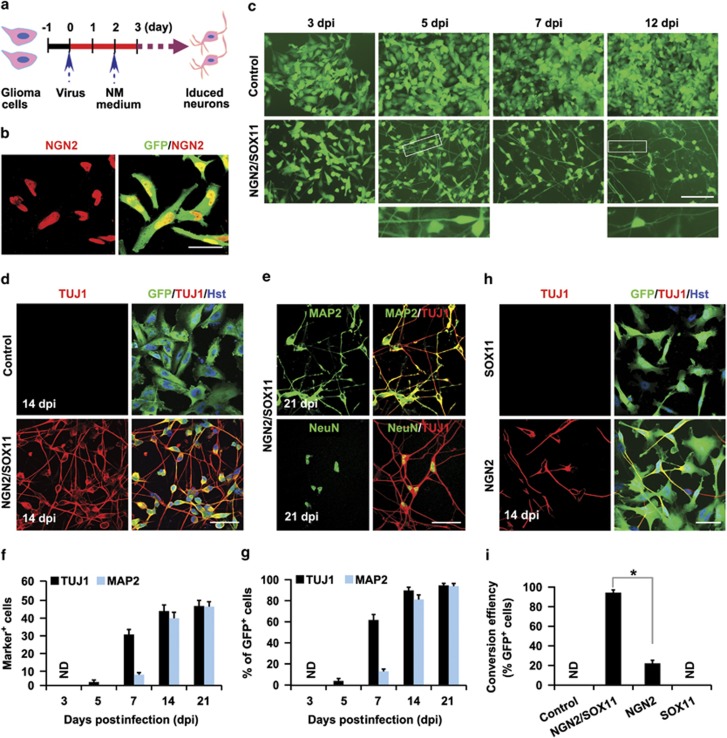
NGN2/SOX11-mediated efficient fate change of human glioma cells. (**a**) Experimental schemes. NM, neuronal induction medium containing forskolin and dorsomorphin. (**b**) Immunocytochemistry confirming NGN2 expression in the virus-transduced U251 human glioma cells. (**c**) Rapid morphological changes of U251 human glioma cells induced by ectopic expression of NGN2/SOX11. GFP-expressing virus was used as a control. Higher magnification views of the boxed regions are also shown. (**d**) TUJ1^+^ neuron-like cells derived from NGN2/SOX11-expressing U251 cells. (**e**) Expression of markers for mature neurons in NGN2/SOX11-converted cells. (**f** and **g**) Quantification of neuronal marker expression in NGN2/SOX11-infected U251 cells during the indicated time course (*n*=20 random fields from triplicate samples; ND, not detected). (**h**) Expression of TUJ1 in U251 cells singly infected with virus expressing NGN2 or SOX11. (**i**) Conversion efficiency normalized to virus-infected cells. TUJ1^+^ cells were quantified from triplicate samples at 14 dpi. *P<0.0001 by Student's *t*-test. Scales: 50 *μ*m (**b**, **d**, **e** and **h**) and 100 *μ*m (**c**)

As our previous work showed that NGN2 and SOX11 can cooperatively reprogram human fibroblasts to postmitotic neurons with high efficiency,^24^ we hypothesized that this reprogramming process might also work on glioma cells. To test this hypothesis, we infected human malignant glioma cells, U251 and U87, with lentivirus-expressing NGN2-IRES-GFP-T2A-SOX11 (NGN2/SOX11) (Figure 1a). Based on the co-expressed green fluorescent protein (GFP), the infection efficiency was estimated to be about 95% (Supplementary Figure S1), although this varied slightly between experiments. Immunocytochemistry showed that NGN2 protein is indeed ectopically expressed in GFP^+^ cells (Figure 1b). At 2 days postinfection (dpi), serum-containing culture medium was switched to forskolin (FSK)- and dorsomorphin (DM)-supplemented neuronal induction medium, which was shown to be essential for neuronal survival and maturation^24^ (Figure 1a). Interestingly, forced expression of NGN2/SOX11 induced glioma cells to rapidly lose their pleomorphic or epithelial morphology and adopt a bipolar appearance as early as 5 dpi (Figure 1c). By 12 dpi, the NGN2/SOX11-infected cells acquired a more complex morphology with multiple neuron-like processes (Figure 1c). Immunocytochemistry showed that such morphological changes were accompanied by robust expression of pan-neuronal markers doublecortin (DCX), TUJ1, microtubule-associated protein 2 (MAP2), and neuron-specific nuclear protein (NeuN; Figures 1d and e; Supplementary Figures S2 and S3A and B). In sharp contrast, cells infected with the control GFP-expressing virus retained their pleomorphic or epithelial morphology and did not express any of the above neuronal markers during the same timeframe (Figures 1c and d; Supplementary Figures S2 and S3A).

A time course analysis of neuronal markers showed that TUJ1 expression could be detected in NGN2/SOX11-expressing cells (indicated by GFP fluorescence) as early as 5 dpi (Figures 1f and g; Supplementary Figures S3C and D). The number of TUJ1^+^ cells sharply increased by 7 dpi. At 14 dpi, 89.8 and 91.2% of NGN2/SOX11-infected U251 and U87 cells were induced to express TUJ1, respectively. The induction of MAP2 expression by NGN2/SOX11 in these glioma cells followed a similar pattern (Figures 1f and g; Supplementary Figures S3C and D). By 21 dpi, >95% of NGN2/SOX11-infected glioma cells had been converted into neuron-like cells, based on the expression of either TUJ1 or MAP2 (Figure 1g; Supplementary Figure S3D). A majority of these cells had multiple and long processes, indicating that they became more morphologically mature over time (Figures 1c–e; Supplementary Figures S3A and B).

### NGN2 and SOX11 synergize in reprogramming human glioma cells

We then determined the relative contributions of these two transcription factors to the neuronal conversion of glioma cells. Approximately 22.2% of glioma cells infected with lentivirus expressing only NGN2 were converted to TUJ1^+^ neuron-like cells, while the expression of SOX11 alone did not generate any of these cells (Figures 1h and i). In sharp contrast, the combinatorial expression of NGN2 and SOX11 converted >95% of virus-transduced human glioma cells into TUJ1^+^ neuron-like cells (Figure 1i). Interestingly, neuronal conversion was not observed in glioma cells infected with lentivirus-expressing ASCL1, neurogenic differentiation 1, or myelin transcription factor 1-like either alone or in combination with SOX11 (Supplementary Figure S4). Together, these data indicate that the synergistic action of NGN2 and SOX11 is required for highly efficient conversion of human glioma cells into neuron-like cells.

### Converted glioma cells exhibit physiological features of neurons

**Figure 2:**
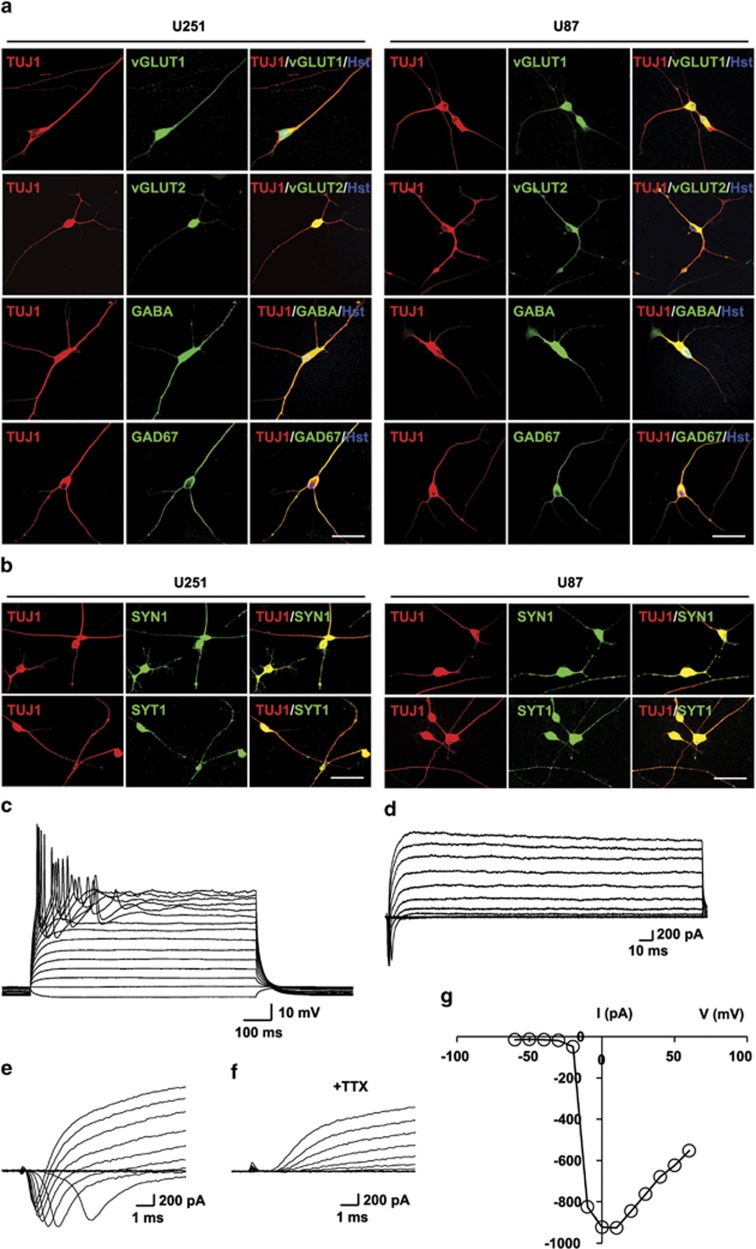
Neuronal features of NGN2/SOX11-reprogrammed human glioma cells. (**a**) Expression of markers for inhibitory (GABA and GAD67) and excitatory (vGLUT1 and vGLUT2) neurons at 21 dpi. (**b**) Expression of the presynaptic markers synapsin-1 (SYN1) and synaptotagmin-1 (SYT1) in the converted cells at 21 dpi. (**c**–**g**) Electrophysiological properties of the converted cells at 52 dpi. These include action potentials induced by depolarizing current injections (**c**), fast inward current and persistent outward current on depolarization (**d**). (**e**–**g**) TTX-sensitive sodium currents elicited at or above −20 mV, with a peak current at ~950 pA. Scales: 60 *μ*m (**a** and **b**)

Immunostaining for neuronal subtypes at 21 dpi showed that >95% of glioma-converted TUJ1^+^ cells were positive for the excitatory neuron marker vesicular glutamate transporter 1 (vGLUT1) or vGLUT2, while <2% of the converted cells expressed GABA (gamma-aminobutyric acid) or GAD67 (67 kDa glutamic acid decarboxylase), which are markers for inhibitory neurons (Figure 2a; Supplementary Figure S5). These data suggest that forced expression of NGN2/SOX11 in glioma cells converted them into a mixed population of neurons, of which a majority is excitatory neurons. By 21 dpi, a majority of the converted TUJ1^+^ cells expressed MAP2 or NeuN, which are markers for mature neurons (Figure 1e; Supplementary Figure S3B). We performed additional immunocytochemical analysis for presynaptic neuronal markers, such as synapsin-1 (SYN1) and synaptotagmin-1 (SYT1). Both SYN1 and SYT1 could be detected in discrete puncta in TUJ1^+^ cells, suggesting the establishment of synaptic termini (Figure 2b).

Electrophysiology was then performed to determine whether glioma cell-derived neurons become functionally mature. When co-cultured with astrocytes for ⩾50 dpi, the NGN2/SOX11-induced neurons showed electrophysiological properties for mature neurons (Figures 2c–g). Action potentials could be elicited by injecting depolarizing current steps (Figure 2c). Depolarizing voltage steps elicited fast inward currents and persistent prolonged outward currents that resemble those from voltage-dependent sodium and potassium channels in primary neurons, respectively (Figures 2d and e). The inward currents were sensitive to treatments with the sodium channel blocker tetrodotoxin (TTX), confirming their sodium current identity (Figure 2f). The analysis of current–voltage (I–V) characteristic showed a peak amplitude close to 1 nA (Figure 2g).

### Neuronal conversion of glioma cells results in cell cycle exit

**Figure 3:**
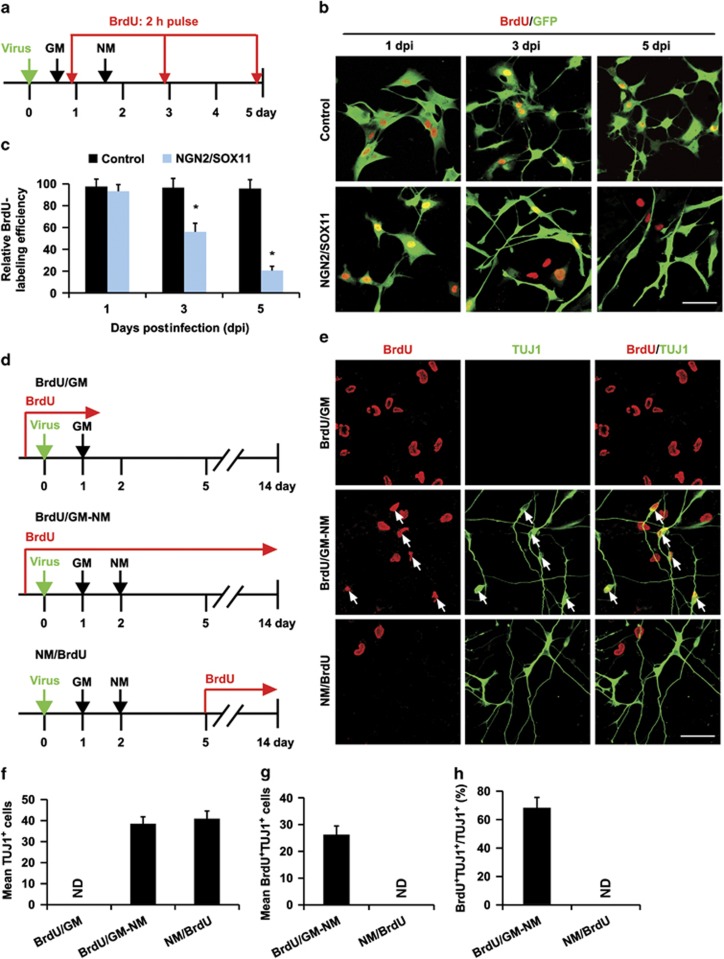
The reprogramming process leads to cell cycle exit. (**a**) The experimental scheme for data presented in panels **b** and **c**. After virus infection, U251 cells were incubated with BrdU for 2 h before immunocytochemistry at the indicated time points. GM, growth medium; NM, neuronal induction medium. (**b** and **c**) Analysis of proliferating cells (*n*=20 randomly selected fields from triplicate samples, *P<0.001 by Student's *t*-test). (**d**) The experimental scheme for data presented in panels **e**–**h**. U251 cells were infected with virus and treated with BrdU for the indicated periods of time. (**e**) Immunocytochemistry showing BrdU-labeled cells. No neuron-like cells were identified under the growth medium (GM) condition. Continuous incubation with BrdU (BrdU/GM-NM) did not affect neuronal survival. Reprogramming by NGN2/SOX11 resulted in rapid inhibition of proliferation (NM/BrdU). Arrows indicate BrdU^+^TUJ1^+^ cells. (**f**–**h**) Quantification of reprogrammed cells and cell proliferation (*n*=20 randomly selected fields from triplicate samples; ND, not detected). Scales: 50 *μ*m (**b** and **e**)

We examined whether neuronal conversion of glioma cells by NGN2/SOX11 changed their growth characteristics, as neurons are normally postmitotic. Two days post infection with lentivirus expressing either the control GFP or NGN2/SOX11, the U251 glioma cells were switched to neuronal induction medium supplemented with FSK and DM. Proliferating cells were pulse-labeled with 5-bromodeoxyuridine (BrdU, 10 *μ*M) for 2 h before immunocytochemical analysis at 1, 3, or 5 dpi (Figure 3a). More than 70% of control GFP- or NGN2/SOX11-infected cells could be labeled by BrdU at 1 dpi (Figures 3b and c). When compared with the controls, 40 and 80% reductions in BrdU labeling of NGN2/SOX11-expressing cells were observed at 3 and 5 dpi, respectively (Figures 3b and c). Accompanying the dramatic reduction of BrdU^+^ cells was the quick adoption of neuron-like elongated morphology of NGN2/SOX11-expressing glioma cells (Figure 3b).

To examine whether glioma-converted neurons were still capable of proliferation, we performed continuous BrdU labeling (Figure 3d). As a control, BrdU was added into the culture medium prior to viral infection. Under this condition, the non-converted (BrdU/GM) or converted (BrdU/GM-NM) cells could be BrdU labeled, indicating that the continuous presence of BrdU did not cause specific loss of labeled cells (Figures 3d–h). In contrast, when BrdU was added into the neuronal induction medium at 5 dpi (NM/BrdU), almost none of the converted cells were labeled, indicating that the reprogramming process leads to exit from the cell cycle (Figures 3d–h).

**Figure 4:**
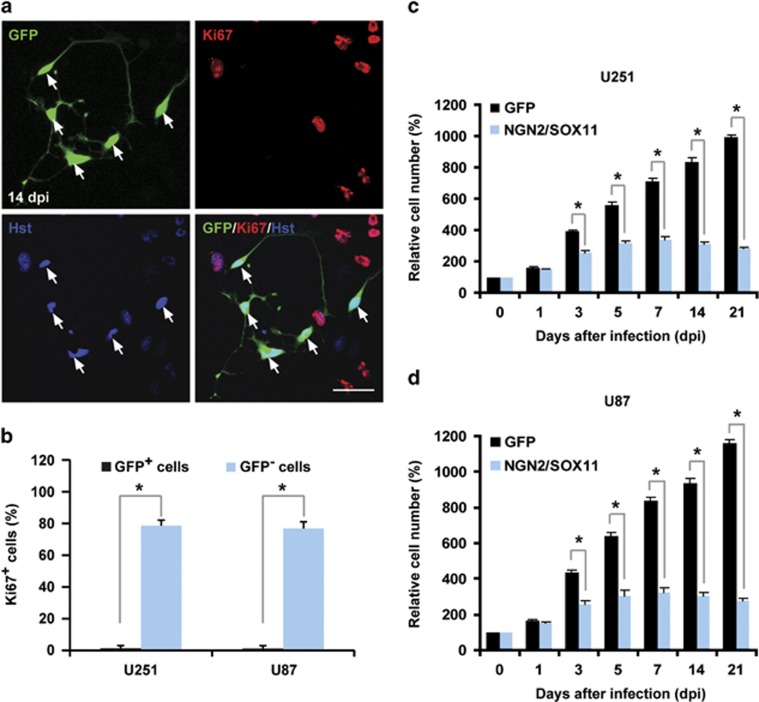
Inhibition of glioma cell proliferation by ectopic NGN2/SOX11. (**a** and **b**) Ki67 staining of U251 cells at 14 dpi. A majority of NGN2/SOX11-expressing cells (GFP^+^) acquired a neuron-like morphology and was Ki67 negative (indicated by arrows). Quantitative data were from 20 randomly selected fields from triplicate samples (*n*=3; *P<0.01 by Student's *t*-test; scale, 50 *μ*m). (**c** and **d**) Quantification of cell numbers at the indicated times after virus infection (*n*=3; *P<0.01 by Student's *t*-test)

Cell proliferation was further examined by immunocytochemistry and cell counting. By 14 dpi, very few NGN2/SOX11-expressing GFP^+^ cells stained positive for Ki67, an endogenous marker for proliferating cells, whereas nearly 80% of non-infected GFP^−^ cells expressed this marker (Figures 4a and b). A time course analysis of cell numbers showed that cell growth reached a plateau at 5 dpi for cells that were infected with virus expressing NGN2/SOX11 (Figures 4c and d). This was in contrast to a continued rapid growth of cells that were infected with the control GFP-expressing virus. Taken together, these data indicate that forced expression of NGN2/SOX11 efficiently induces neuronal conversion of glioma cells and that it leads to rapid cycle exit and inhibition of cell proliferation and growth.

### Loss of tumorigenic potential of NGN2/SOX11-expressing glioma cells

**Figure 5:**
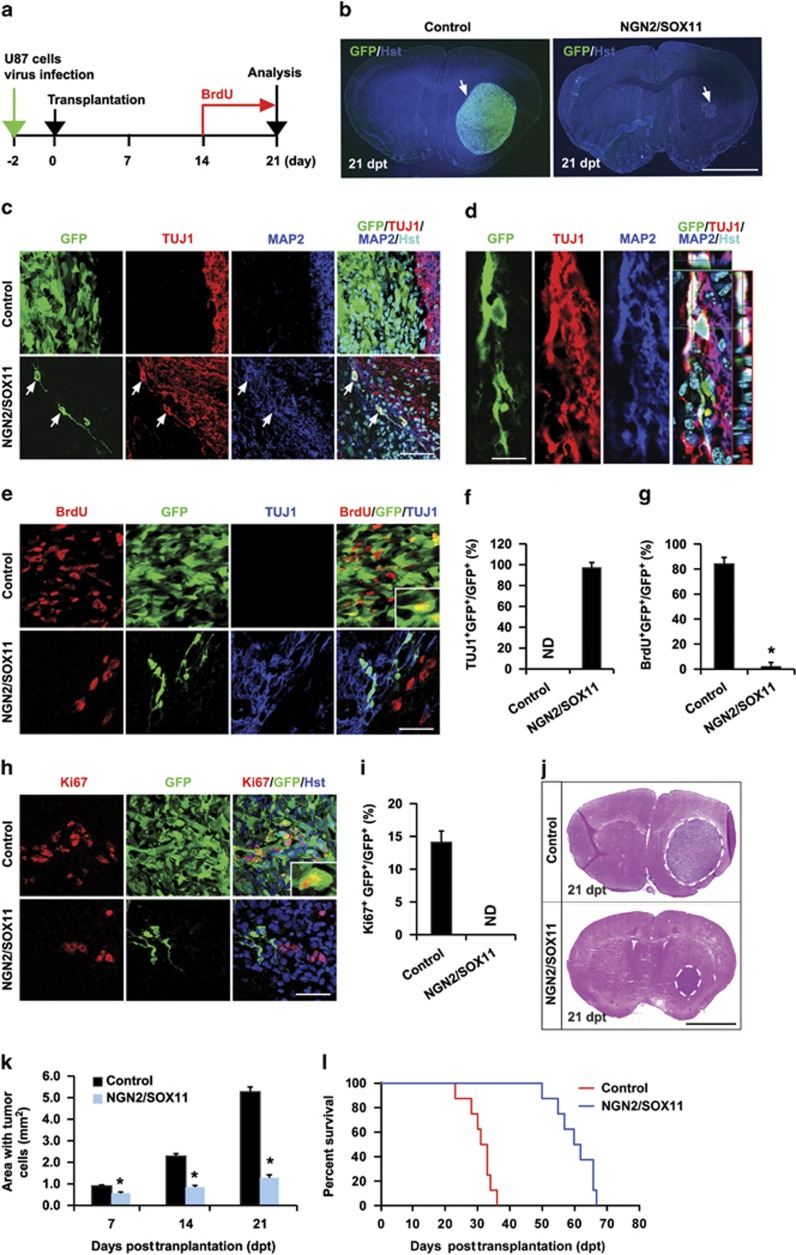
Loss of tumorigenicity of reprogrammed human glioma cells. (**a**) The experimental scheme for data presented in panels **b**–**k**. Animals were intraperitoneally injected with BrdU to label proliferating cells. (**b**) Coronal brain sections showing tumor formation (indicated by arrows) at 21 days posttransplantation (dpt). GFP expression indicates virus-infected cells. (**c** and **d**) Detection of neuron-like cells (shown by arrows) in brains transplanted with NGN2/SOX11-infected U87 cells at 21 dpt. A confocal analysis of the glioma-converted neuron-like cells is shown in panel **d**. (**e**–**i**) Lack of proliferation of NGN2/SOX11-expressing human glioma cells *in vivo* (*n*=3 for each group; *P<0.01 by Student's *t*-test). (**j** and **k**) A time course analysis of tumor formation in mice transplanted with U87 cells that were infected with control or NGN2/SOX11 virus. Tumor mass (outlined by dashed lines) was quantified from histologically stained coronal brain sections (*n*=3 for each group; *P<0.01 by Student's *t*-test). (**l**) Kaplan–Meier survival curve of mice transplanted with U87 cells that were infected with the indicated virus (*n*=8 for each group; *P*=0.0001 by log-rank test). Scales: 1 mm (**b** and **j**), 50 *μ*m (**c**, **e,** and **h**), and 15 *μ*m (**d**)

Rapid cell cycle exit of NGN2/SOX11-expressing glioma cells suggests that these cells might also lose their ability to generate tumors *in vivo*. To examine this possibility, we performed orthotopic cell transplantation experiments. Human U87 glioma cells were infected with lentivirus expressing either the control GFP or NGN2/SOX11. The infection efficiency was estimated at 95% for both of these two groups. At 2 dpi, 5 × 10^5^ dissociated cells were stereotactically injected into the striatum of NOD scid gamma (NSG) mice.^34^ Cellular identity and tumorigenicity of the engrafted human glioma cells were evaluated at 21 days posttransplantation (Figure 5a). As shown in Figure 5b, marked tumor masses composed of densely packed GFP^+^ cells were readily detectable in the brains transplanted with the control GFP-expressing U87 cells. This was in stark contrast to small tumor masses that were observed in brains injected with NGN2/SOX11 virus-infected glioma cells (Figure 5b). These latter tumors were essentially formed by GFP-negative cells, indicating that the residual uninfected glioma cells eventually grew up and became tumorigenic. However, a few GFP^+^ cells were also observed in brains transplanted with NGN2/SOX11-expressing U87 cells (Figure 5c). Very interestingly, immunohistological analysis showed that a majority of these GFP^+^ cells acquired neuron-like morphology and expressed the pan-neuronal markers TUJ1 and MAP2 (Figures 5c, d, and f). On the other hand, none of the control GFP-infected U87 cells showed neuronal properties *in vivo* (Figure 5c). These data indicate that NGN2/SOX11-expressing glioma cells can be converted *in vivo* to neuron-like cells.

The lack of tumorigenic GFP^+^ cells in the brains engrafted with NGN2/SOX11-expressing glioma cells suggested that they stopped proliferation after transplantation. To examine this in detail, we labeled proliferating cells with BrdU (i.p., 100 mg/kg, once per day) for 1 week prior to killing (Figure 5a). More than 80% of control GFP-expressing transplanted U87 cells incorporated BrdU (Figures 5e and g). Around 14% of these control cells were also stained positive for Ki67 (Figure 5i). In sharp contrast, almost none of the NGN2/SOX11-expressing cells (indicated by GFP coexpression) were either labeled by BrdU or stained positive for Ki67, although BrdU^+^ or Ki67^+^ cells were still detectable in GFP-negative cells within the same brain sections (Figures 5e, g and h). These data indicate that NGN2/SOX11-expressing glioma cells stopped proliferation after transplantation.

As GFP-negative cells were still proliferating in the brains transplanted with glioma cells that were infected with NGN2/SOX11-expressing virus, we examined the time course of tumor growth by measuring areas containing human glioma cells at 7, 14, and 21 days posttransplantation (Figures 5j and k). The control GFP-infected U87 glioma cells rapidly expanded tumors with a feature of explosive growth (Figures 5j and k). Although tumors were eventually formed in the brains engrafted with glioma cells that were infected with NGN2/SOX11 virus, they grew slowly and were largely formed by noninfected GFP-negatvie cells (Figures 5j and k). As a consequence, the mice transplanted with these cells survived significantly longer than those engrafted with glioma cells that were infected with the control GFP virus (Figure 5l).

### *In vivo* reprogramming of malignant glioma cells impedes tumor growth

**Figure 6:**
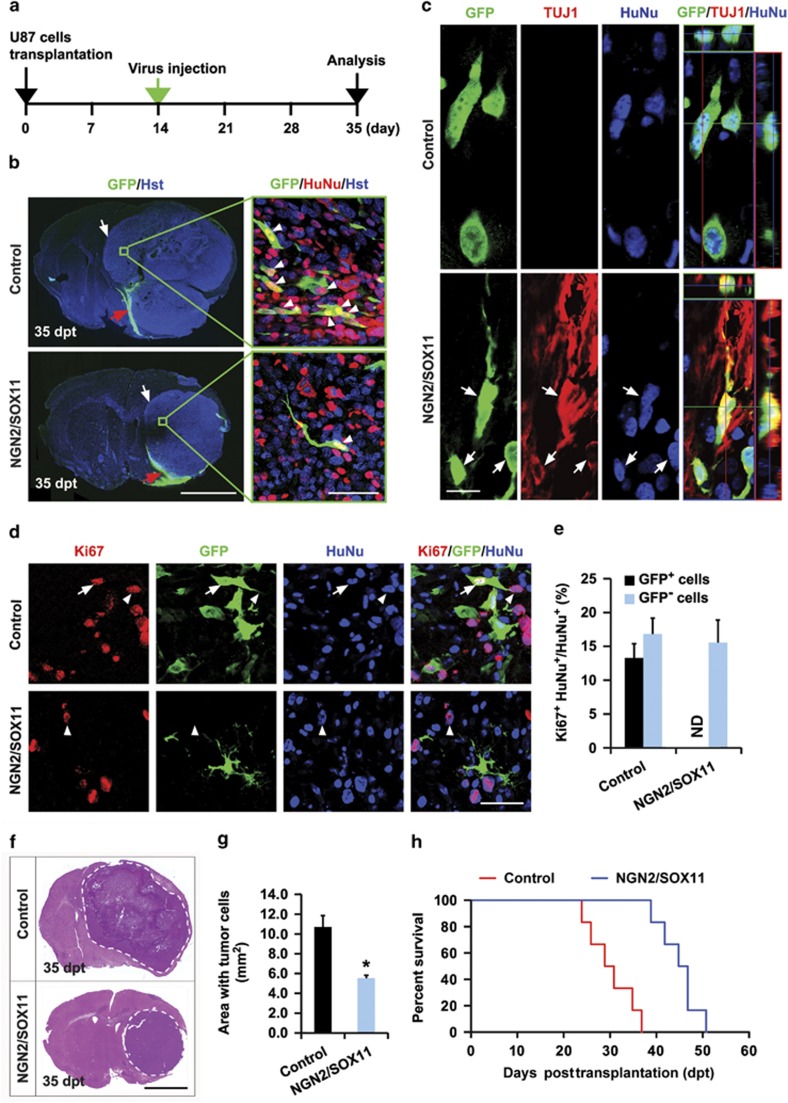
*In vivo* reprogramming of human glioma cells prolongs mouse survival. (**a**) The experimental scheme for data presented in panels **b**–**g**. (**b** and **c**) Ectopic expression of NGN2/SOX11 results in neuron-like cells from previously transplanted human glioma cells. (**b**) Lower magnification views of coronal brain sections across the tumor mass (indicated by white arrows). Injected virus also infected some endogenous brain cells (shown by GFP expression and indicated by red arrows), in addition to the transplanted U87 cells (indicated by GFP and human nuclei (HuNu) and shown by arrow heads). dpt, days posttransplantation. (**c**) Confocal analysis of the virus-infected U87 cells at 35 dpt. Glioma cell-converted neuron-like cells (indicated by arrows) were only detected in NGN2/SOX11-expressing cells. (**d** and **e**) Expression of NGN2/SOX11 in pretransplanted U87 cells results in proliferation inhibition. Proliferating human glioma cells with and without virus infection are indicated by arrows and arrowheads, respectively. Quantification data were from 20 randomly selected fields from triplicate samples. ND, not detected. (**f** and **g**) Ectopic NGN2/SOX11 inhibits tumor growth of previously transplanted U87 cells. Area with tumor cells was estimated from histological coronal brain sections at 35 dpt (*n*=4 for each group; *P<0.01 by Student's *t*-test). (**h**) Kaplan–Meier survival curve of mice transplanted with U87 cells and injected with the indicated virus (*n*=6 mice for each group; *P*=0.0005 by log-rank test). Scales: 1 mm (lower magnification views in **b** and **f**), 50 *μ*m (higher magnification views in **b** and **d**), and 15 *μ*m (**c**)

To determine whether reprogramming the fate of glioma cells has any therapeutic potential for brain tumors, we examined the effect of ectopic expression of NGN2/SOX11 on the growth of preexisting tumors (Figure 6a). Brain tumors were initiated through transplantation of 5 × 10^5^ U87 cells into the striatum of NSG mice. At 2 weeks posttransplantation, the mice were randomized and stereotactically injected with lentivirus expressing either GFP or NGN2/SOX11 into the same location as the transplanted cells. A subset of mice (two from each group) was examined immediately after viral injections, and mice from the different groups had a similar tumor masses at this time (Supplementary Figure S6). Five days after viral injections, another subset of mice (two from each group) were killed to determine viral infection efficiency of the transplanted glioma cells, which were identified by staining for human nuclei protein. The infection efficiency was estimated at around 40% for both the control GFP and NGN2/SOX11 virus (Supplementary Figure S7). When examined at 5 weeks posttransplantation, very interestingly, some of the NGN2/SOX11-infected (indicated by GFP coexpression) human glioma cells acquired a neuron-like morphology and expressed the pan-neuronal marker TUJ1 (Figures 6b and c). However, a majority of the NGN2/SOX11-infected human glioma cells seemed to undergo cell death, indicated by pyknosis or fragmented cell bodies (Supplementary Figure S8). In contrast, the control GFP virus-infected human glioma cells neither showed morphological changes nor expressed the neuronal marker TUJ1 *in vivo* (Figures 6b and c).

Proliferation of the engrafted human glioma cells was analyzed at 5 weeks posttransplantation, which was 3 weeks after viral injection. About 13% of the control GFP-infected glioma cells stained positive for Ki67, whereas no Ki67 was detected in NGN2/SOX11 virus-infected glioma cells (Figures 6d and e). As internal controls for immunohistochemistry, 16.8 and 15.5% of the non-infected (GFP^−^) human glioma cells were detected in mice injected with lentivirus expressing the control GFP and NGN2/SOX11, respectively. These data indicate that *in vivo* overexpression of NGN2/SOX11 in tumors leads to rapid inhibition of glioma cell proliferation. Accordingly, tumor burden was significantly reduced in mice injected with the NGN2/SOX11-expressing virus when examined at 35 days posttransplantation (Figures 6f and g). Although non-transduced glioma cells quickly expanded and eventually led to death of the engrafted mice, Kaplan–Meier survival analysis showed that direct injection of NGN2/SOX11-expressing virus into tumor-bearing mice significantly extended their lifespan (Figure 6h).

## Discussion

Uncontrolled cell proliferation and invasion are the major cause of the deadly nature of human gliomas. Here we explored a potential therapeutic approach for brain tumors by changing the fate of glioma cells through transcription factor-mediated reprogramming. Our results demonstrate that ectopic expression of NGN2 and SOX11 can synergistically and efficiently convert human glioma cells into postmitotic neuron-like cells, which ultimately lose their tumorigenic capacity. Importantly, acute expression of these two factors in tumor-bearing mice also halts proliferation of glioma cells *in vivo* and leads to greatly reduced tumor burden and prolonged survival.

Conversion of tumor-initiating cells into terminally differentiated cell types represents a novel therapeutic strategy for tumors.^7,23,28,32^ The conversion rate will be a critical determinant for this approach to be successful. Recently, it was shown that the forced expression of *Ascl1*, *Brn2* and *Ngn2* in glioma cells can convert them into neurons with an efficiency of 20–40%.^23^ With a different and simpler combination of factors, our current study demonstrates that the efficiency could be significantly improved, with 95% of virus-infected human glioma cells converted to neuron-like cells. Such highly efficient cell fate conversion requires synergy between NGN2 and SOX11, as the efficiency for NGN2 alone is only 22% and SOX11 alone has no effect on cell fate change. Interestingly, neuron-like cells begin to appear as early as 5 dpi of glioma cells with NGN2/SOX11-expressing virus. By 14 dpi, a majority of the infected cells express markers and exhibit morphology for neurons. These data clearly indicate that human glioma cells are amenable to reprogramming.

Although the glioma-converted neurons are heterogeneous, the predominant subtype is glutamatergic, with >95% of them expressing the markers vGlut1 and vGlut2. The reprogramming process is accompanied by the transient expression of DCX, a microtubule-associated protein that is highly expressed in neuroblasts and immature neurons during development and in neurogenic regions of the adult brain.^35,36^ DCX is not detectable in human glioma cells,^37,38^ and its expression is positively associated with patient survival.^39^ Ectopic expression of DCX was shown to inhibit glioma cell proliferation and invasion by interacting with spinophilin/neurabin II, a tumor suppressor, and PP1, a serine/threonine protein phosphatase.^37,38^ The induction of DCX expression by NGN2/SOX11 in human glioma cells might contribute to their growth arrest.

When human glioma cells were transplanted into the mouse striatum 2 dpi with NGN2/SOX11-expressing lentivirus, they could be converted into neuron-like cells *in vivo*. Importantly, direct injection of NGN2/SOX11-expressing lentivirus into a preexisting tumor mass also resulted in the appearance of neuron-like cells. These data are consistent with previous observations that the *in vivo* microenvironment is suitable for cellular reprogramming.^20,21,33^ However, a majority of the glioma-converted neuron-like cells cannot survive *in vivo*, suggesting that additional neurotrophic factors might be required for long-term survival and maturation of the converted neurons, as is the case for induced neuroblasts from adult resident astrocytes.^21,22^ The limited survival of glioma-converted neurons *in vivo* might be therapeutically beneficial as these neurons may form abnormal neural circuits with preexisting neurons and interfere with normal brain function.

Ectopic expression of NGN2/SOX11 in glioma cells results in inhibition of cell proliferation. Nonetheless, the remaining non-infected glioma cells eventually grow up and become tumorigenic. This explains why brain tumors developed in mice engrafted with human glioma cells that were infected with virus expressing NGN2/SOX11. The most interesting finding is that direct injection of NGN2/SOX11-expressing virus into preexisting brain tumors can reprogram glioma cells *in vivo* and lead to prolonged survival. This result suggests that *in vivo* cellular reprogramming could be a promising therapeutic approach for aggressive brain tumors. However, significantly improved efficiency and glioma targeting specificity of viral transduction will be essential for therapeutic purposes. This could be achieved using a different virus-mediated gene delivery system,^40^ multiple site injections,^41^ continuous infusion of larger volume using a cannula,^42^ and cell type-specific promoters.^40^ Notwithstanding these limitations, results of this proof-of-concept study will provide a solid foundation for future improvements and the ultimate establishment of a potentially paradigm-shifting therapeutic strategy for incurable malignant human gliomas. This strategy could be combined with neurosurgery, chemo- and radio-therapy to achieve the best outcome for brain tumor patients.

## Materials and Methods

### Plasmids construction and virus production

After PCR amplification, cDNAs encoding the human NGN2 and SOX11 were subcloned into a third-generation lentiviral vector (pCSC-SP-PW-IRES/GFP) to generate pCSC-SP-PW-NGN2-IRES-GFP-T2A-SOX11 (abbreviated NGN2/SOX11). The co-expressed GFP was used to visualize virus-infected cells. The parental plasmid was used as a control. Replication-deficient lentivirus was produced in HEK293T cells by transient transfections with the lentiviral vector and packaging plasmids (pMDL, VSV-G, and pREV).^43^ Lentivirus was collected and concentrated by ultracentrifugation at 112 000 × *g* for 2 h at 4 °C. Concentrated virus was stored at −80 °C until use.

### Cell culture and virus infection

Human glioma cell lines, U251 and U87, were cultured in DMEM supplemented with 10% fetal bovine serum. Cells were seeded on gelatin- or Matrigel-coated culture vessels with or without glass coverslips. The following day, they were infected with the indicated lentivirus in the presence of 6 *μ*g/ml polybrene. After overnight incubation, culture media were refreshed. One day later, the cells were then switched to neuronal induction medium, which consists of DMEM:F12:Neurobasal (2 : 2 : 1), 0.8% N-2 (Invitrogen, Carlsbad, CA, USA), and 0.4% B-27 (Invitrogen). In addition, forskolin (FSK, 10 *μ*M) and dorsomorphin (DM, 1 *μ*M) were added to the above induction medium. Induction medium was half-changed every other day. TUJ1^+^ cells with a neuronal morphology, indicated by a round or pyramidal soma with a thin process at least three times longer than the cell body, were counted at the indicated time points. The conversion efficiency was estimated by dividing the total number of neuron-like cells by the number of virus-infected cells (indicated by GFP) or initially seeded cells.

### Immunofluorescence and histological staining

For immunocytochemistry, cells cultured on coverslips were fixed with 4% paraformaldehyde in PBS for 20 min at room temperature. For immunohistochemistry, mice were killed by CO_2_ overdose and fixed by intracardial perfusion with 4% paraformaldehyde in PBS. Brains were surgically dissected out, postfixed overnight, and cryoprotected with 30% sucrose at 4 °C for 48 h. Coronal brain sections were collected at 40-*μ*m thickness on a cryostat. Fixed cells or brain sections were permeabilized and blocked with 0.2% Triton X-100 and 3% BSA in 1 × PBS for 1 h, followed by overnight incubations at 4 °C with the primary antibodies listed in Supplementary Table S1. Alexa Fluor 488-, 594-, or 647-conjugated corresponding secondary antibodies from Jackson ImmunoResearch (West Grove, PA, USA) were used for indirect fluorescence. Nuclei were counterstained with Hoechst 33342. Images were captured with an Olympus fluorescence microscope (Olympus America, Center Valley, PA, USA) or a Zeiss LSM 510 confocal microscope (Zeiss, Oberkochen, Germany). In addition, for *in vivo* quantification, cell numbers were estimated from a series of every eighth coronal brain section from each animal. Tumor sizes were quantified from 10 representative 40-*μ*m-thick serial coronal brain sections that were stained with hematoxylin and eosin (HE).

### BrdU labeling and cell proliferation assays

Proliferating cells in culture were labeled by incubation with 10 mM BrdU (Sigma) for 2 h or as indicated. Proliferating cells in animals were labeled by intraperitoneal injections of BrdU (100 mg/kg body weight, once daily) for the indicated durations. BrdU incorporation was detected by fluorescent staining using an anti-BrdU antibody (rat, 1 : 500, Accurate Chemical, Westbury, NY, USA). Briefly, paraformaldehyde-fixed cells or brain sections were treated with 2 M HCl for 30 min at 37 °C, rinsed in 0.1 M boric acid for 10 min, and incubated with blocking solution (0.2% Triton X-100 and 3% BSA in 1 × PBS) for 1 h. This was then followed by sequential incubations with blocking solutions containing primary and secondary antibodies. Proliferating cells were also detected with an antibody for Ki67 (rabbit, 1 : 500, Novocastra, Newcastle, UK). Cell numbers were counted at the indicated time points after infection of 4 × 10^4^ glioma cells with lentivirus. Colony-formation assays were performed as previously described.^23^ Briefly, glioma cells were infected with lentivirus in a 10 cm dish. Three days later, they were replated into a six-well plate at a low cell density (2 × 10^3^ cells/well). The number and size of the resulting colonies were determined 2 weeks postreplating.

### Electrophysiology

The human glioma-converted neurons were co-cultured with astrocytes on glass coverslips for 6–8 weeks before electrophysiological analysis. The converted cells were identified using infrared differential interference contrast (IR-DIC) and the coexpressed GFP reporter under epifluorescence. Whole-cell patch clamp recordings were performed at 25 °C (room temperature) in a submersion chamber containing Tyrode bath solution (150 mM NaCl, 4 mM KCl, 10 mM HEPES, 10 mM Glucose, 3 mM CaCl_2_, and 2 mM MgCl_2_ at pH 7.4 and 300 mOsm). Recording pipettes (approximately 5–7 MΩ) were filled with an intracellular solution (0.2 mM EGTA, 130 mM K-Gluconate, 6 mM KCl, 3 mM NaCl, 10 mM HEPES, 4 mM ATP-Mg, 0.4 mM GTP-Na, and 14 mM phosphocreatine-di(Tris) at pH 7.2 and 285 mOsm). Series and input resistance were measured under voltage clamp with a 400-ms, 10-mV step from a −60-mV holding potential (filtered at 30 kHz, sampled at 50 kHz). Cells were used for analysis only if the series resistance was <30 MΩ and was stable throughout the experiment. Input resistance ranged from 0.2 to 2 GΩ. All recordings were obtained with a MultiClamp 700B amplifier (Molecular Devices, Sunnyvale, CA, USA). Currents were filtered at 2 kHz, acquired, and digitized at 10 kHz using Clampex10.3 (Molecular Devices). Action potentials were recorded under current clamp and elicited by a series of current injections ranging from −20 to +200 pA with a 20-pA increment and a 400-ms duration. Sodium currents were recorded under voltage clamp in response to a series of voltage steps ranging from −60 to +60 mV with a 10-mV increment and a 100-ms duration according to standard protocols. TTX (1 mM), a voltage-dependent Na^+^ channel blocker, was then applied to the chamber for 8 min, and the voltage step was repeated to observe the TTX-sensitive currents. In all voltage clamp recordings, cells were clamped at −60 mV except during the voltage step protocol. Data analysis was performed in Clampfit 10.3 (Molecular Devices).

### Orthotopic cell transplantation and stereotactic virus injection

NSG mice^34^ were purchased from the Jackson Laboratories. All mice were housed under a 12-h light/dark cycle and had *ad libitum* access to food and water in the UT Southwestern animal facility. All experimental procedures and protocols were approved by the Institutional Animal Care and Use Committee at UT Southwestern. The indicated U87 cells (5 × 10^5^ cells in 2 *μ*l) were injected into the right striatum of 2–3-month-old NSG mice using a 25-gauge needle. Injection coordinates were: anterior/posterior, +1.0 mm; medial/lateral, +2.0 mm; and dorsal/ventral from skull, −3.0 mm. For direct *in vivo* infection of previously transplanted human glioma cells, 2 *μ*l of virus (0.5–2 × 10^9^ cfu/ml) was injected with a 33-gauge needle and a 5-*μ*l Hamilton syringe along the needle track formed by the earlier cell transplantation under the same stereotactic coordinates. Kaplan–Meier analysis was used to evaluate the survival curve of the transplanted mice.

### Statistical analysis

Data are presented as mean±S.D. Except for mouse survival experiments, all other data were analyzed using the unpaired Student's *t*-test. The Kaplan–Meier survival curves were determined by GraphPad Prism v5.0 (GraphPad Software, Inc., San Diego, CA, USA) and the log-rank test. Differences were considered significant at *P*<0.05.

## Abbreviations

ASCL1: achaete-scute complex homolog 1
BrdU: 5-bromodeoxyuridine
DCX: doublecortin
DM: dorsomorphin
FSK: forskolin
GABA: gamma-aminobutyric acid
GAD67: 67 kDa glutamic acid decarboxylase
GFP: green fluorescent protein
HE: hematoxylin and eosin
MAP2: microtubule-associated protein 2
NeuN: neuron-specific nuclear protein
NEUROD1: neurogenic differentiation 1
NGN2: neurogenin 2
NSG: NOD scid gamma
SOX11: sex-determining region Y-box 11
SYN1: synapsin-1
SYT1: synaptotagmin-1
TTX: tetrodotoxin
TUJ1: class III beta-tubulin
vGLUT: vesicular glutamate transporter
