# Supplementary material for: Reprogramming the fate of human glioma cells to impede brain tumor development
Source: Cell Death Dis. 2014 Oct 16;5(10):e1463–. doi: 10.1038/cddis.2014.425 (PMC4649522; doi:10.1038/cddis.2014.425)
Supplement: Supplementary Information [file cddis2014425x1.pdf]

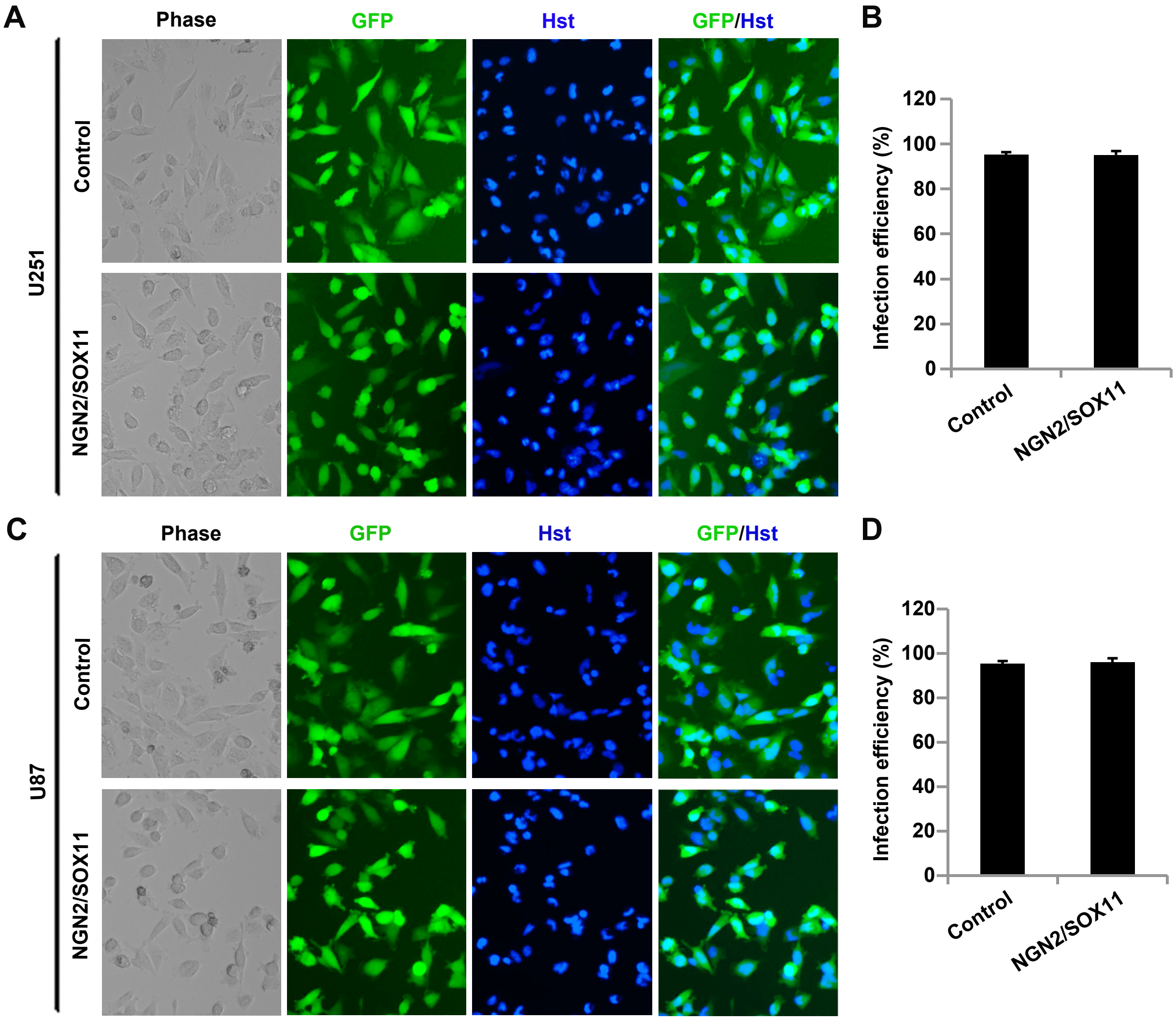

Supplementary Figure 1. Infection efficiency of human glioma cells. (A-D) U251 and U87 cells were infected with the indicated lentivirus and were analyzed 48 h later. The infection efficiency was determined by dividing the number of GFP^{+} cells by the number of Hst^{+} cells. Virus expressing GFP alone was used as controls.

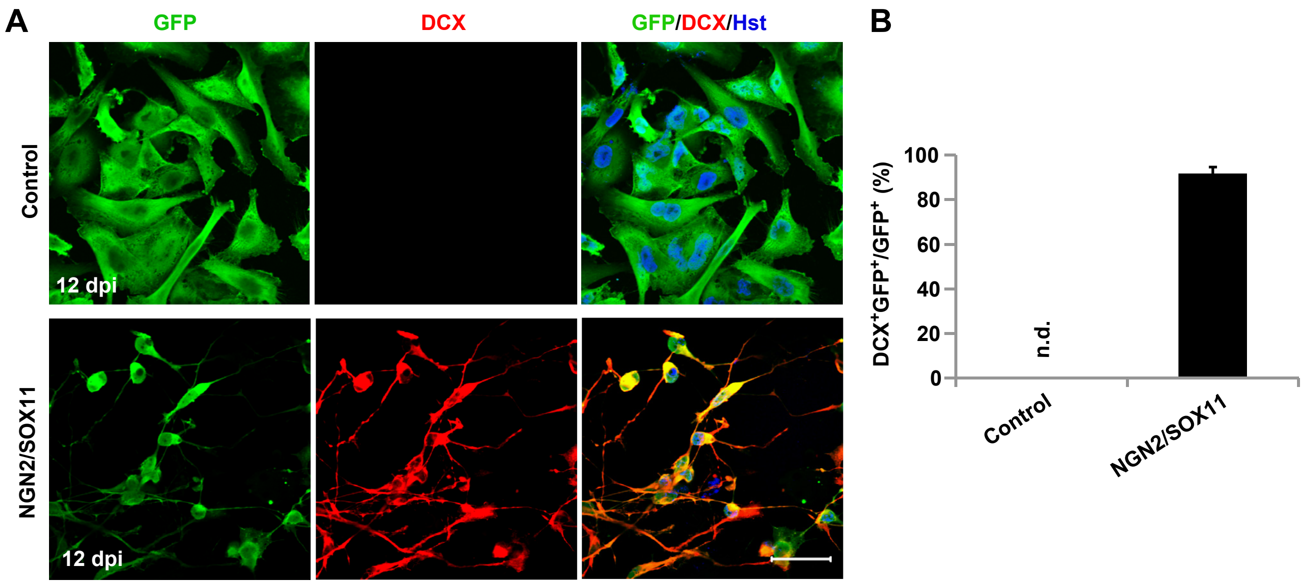

Supplementary Figure 2. Induction of DCX expression by NGN2/SOX11 in human glioma cells. **(A)** Immunocytochemistry showing the expression of DCX at 12 dpi. Scale, 50 μm. **(B)** Quantification of DCX^+ cells (n=20 random fields from triplicate samples; n.d., not detected).

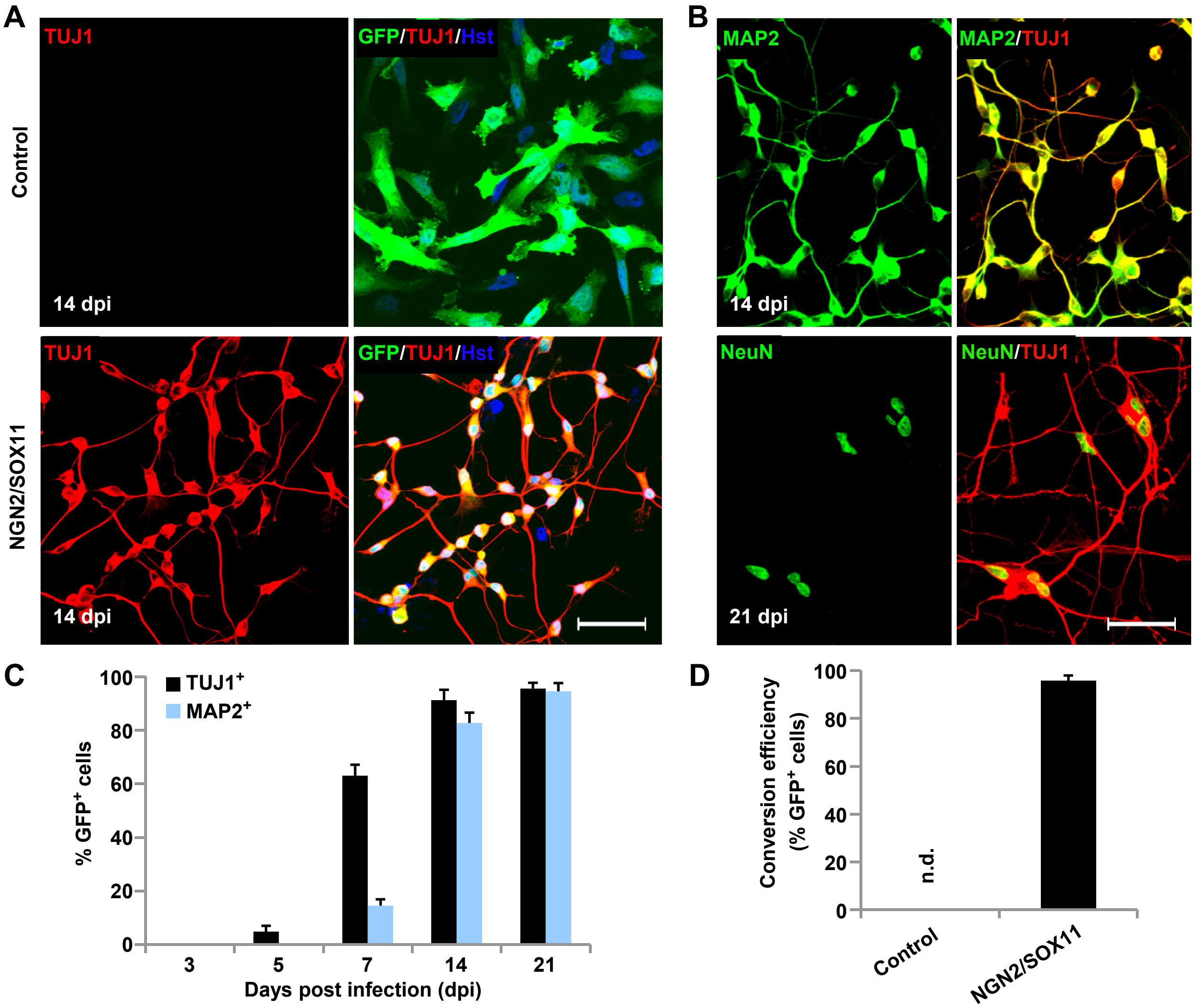

Supplementary Figure 3. Reprograming the fate of U87 cells by NGN2/SOX11. (A) Immunocytochemistry showing NGN2/SOX11 but not the control GFP efficiently converts U87 cells to $TUJ1^+$ cells. Scale, 50 µm. **(B)** NGN2/SOX11-reprogrammed cells express the mature neuronal markers MAP2 and NeuN. Scale, 50 µm. **(C)** Quantification of cells expressing the indicated neuronal markers (n=20 random fields from triplicate samples; n.d., not detected). **(D)** Conversion efficiency. The total number of $TUJ1^+$ cells from triplicate samples was quantified at 14 dpi. This number was then normalized to the number of virus-infected, GFP^+ cells. n.d., not detected.

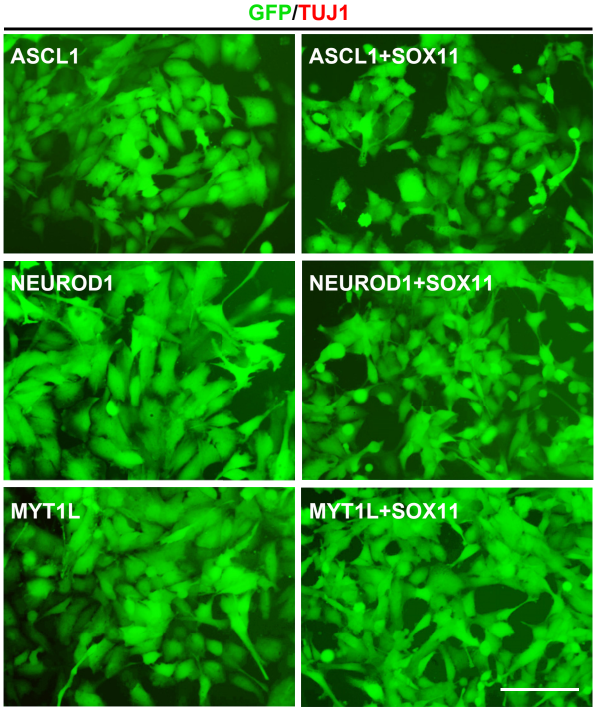

Supplementary Figure 4. ASCL1, NEUROD1, or MYT1L alone or in combination with SOX11 failed to reprogram human glioma cells. Immunohistochemistry showing that $TUJ1^{+}$ neuron-like cells were not detected 10 days post infection of U251 cells with virus expressing the indicated factors. Virus-transduced cells are indicated by the co-expressed GFP marker.

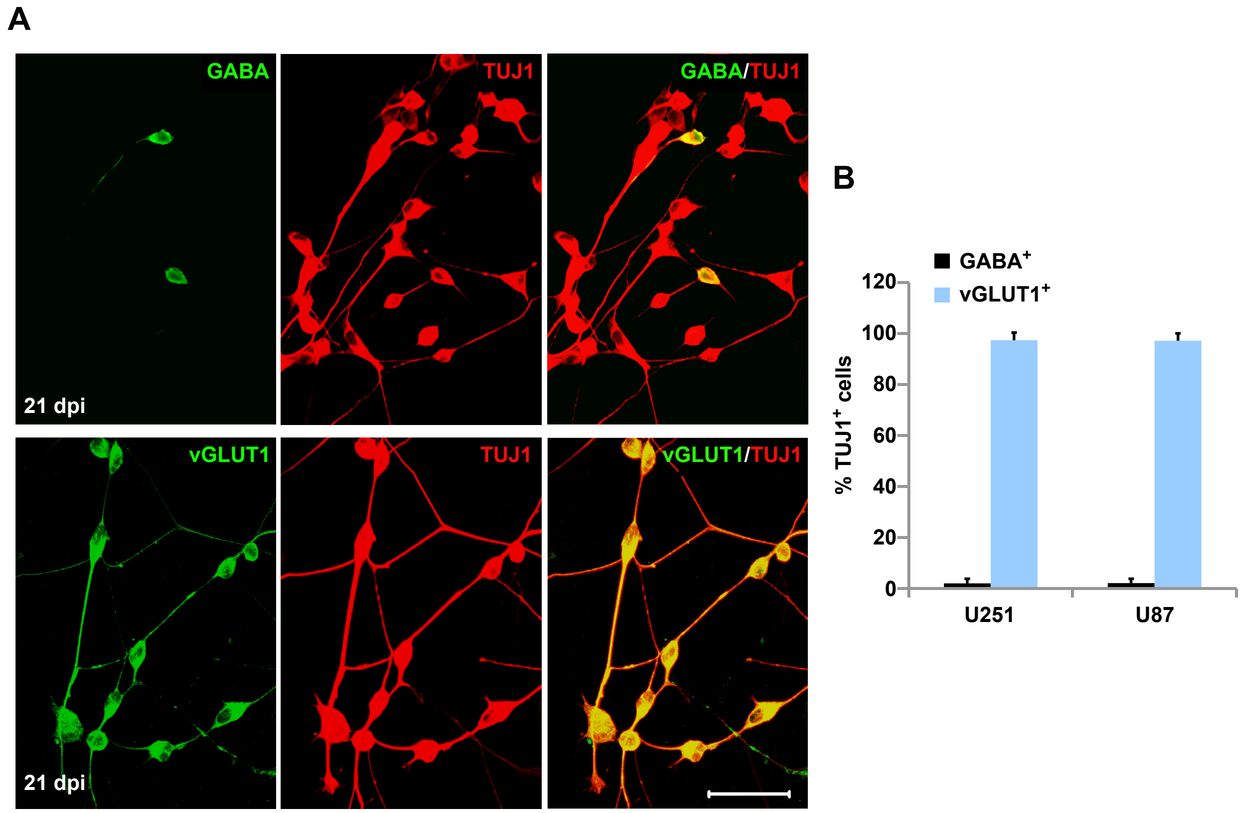

Supplementary Figure 5. NGN2/SOX11 mainly converts human glioma cells to excitatory neurons. (A) Immunocytochemistry showing the expression of markers for inhibitory ($GABA^+$) or excitatory ($vGLUT1^+$) neurons in converted U251 cells. Scale, 50 µm. **(B)** Quantification (n=20 random fields from triplicate samples).

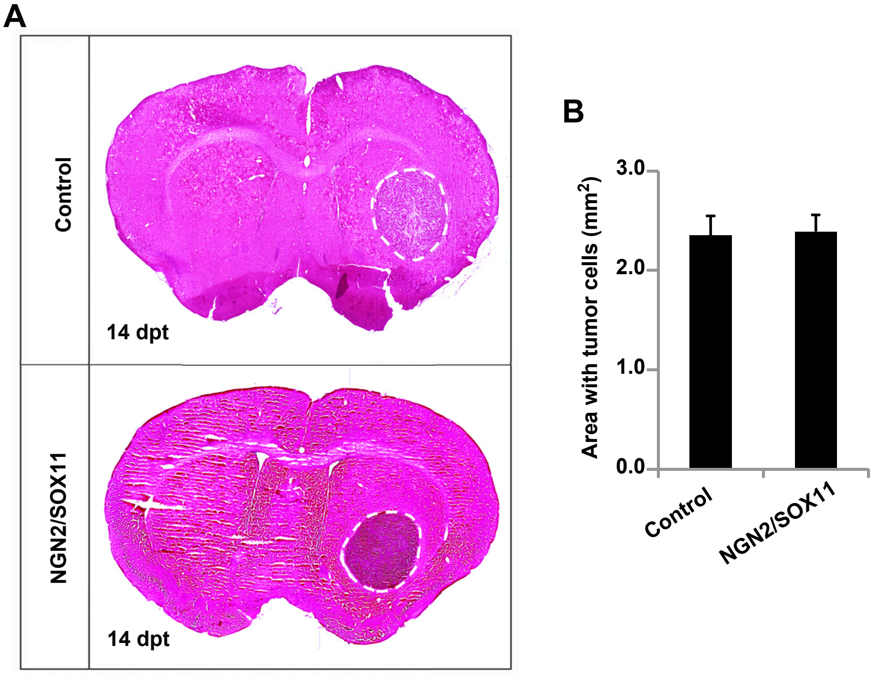

Supplementary Figure 6. Mice with comparable tumor masses were used for virus injection. (A, B) Mice were transplanted with U87 glioma cells and were randomly divided into two groups. Prior to virus injections, a subgroup was analyzed for tumor mass at 14 days post transplantation (dpt) (n=2 mice per subgroup).

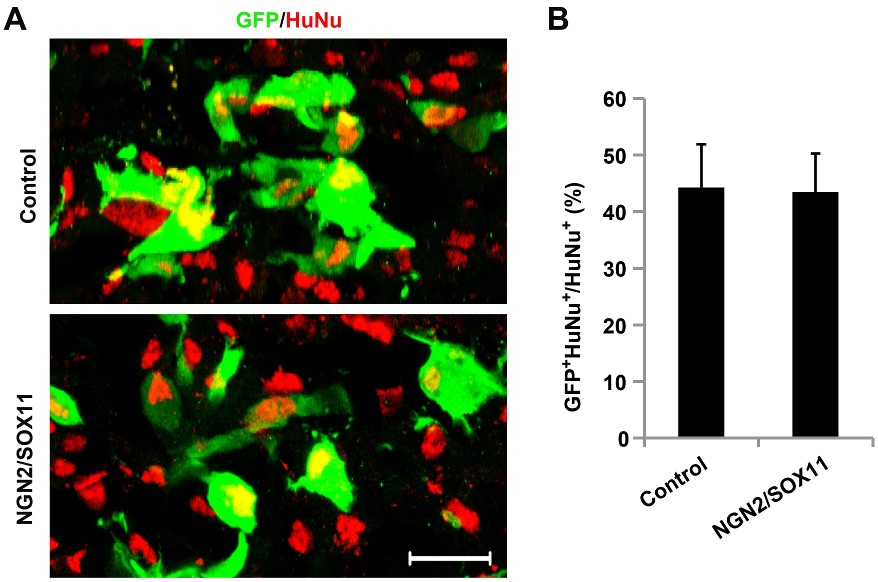

Supplementary Figure 7. Comparable in vivo infection efficiency. (A, B) Mice with pre-transplanted U87 cells were injected with the indicated virus at 14 dpt. Five days after virus injection, a subgroup was analyzed for virus-infected cells (indicated by the co-expressed GFP). The infection efficiency was calculated by dividing the number of GFP^+HuNu^+ cells by the number of $HuNu^+$ cells (n=2 mice per subgroup; Scale, 20 μm).

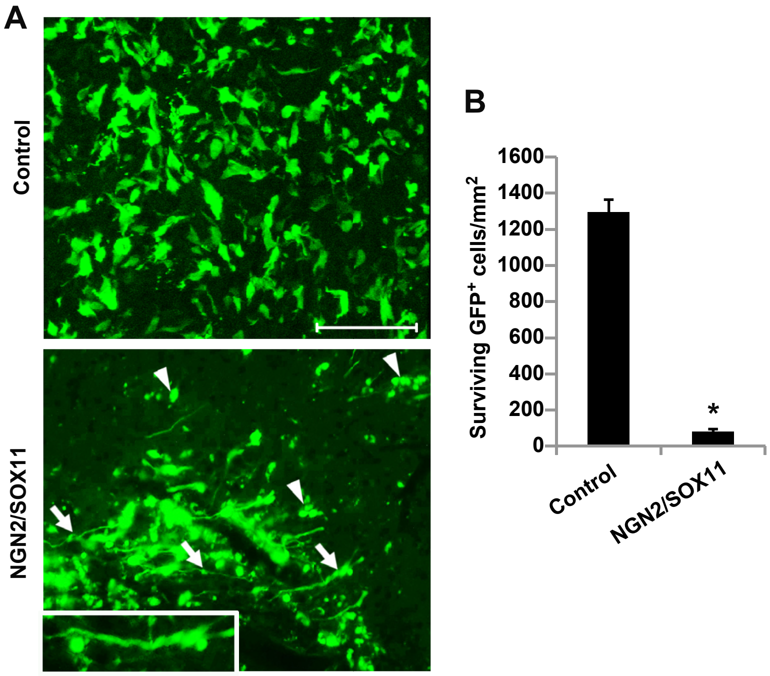

Supplementary Figure 8. A majority of glioma-converted neurons cannot survive in vivo. (A, B) Mice with pre-transplanted U87 cells were injected with the indicated virus at 14 dpt. Three weeks after virus injection, the surviving infected cells (indicated by GFP) were quantified. Arrows show neuron-like cells, whereas arrowheads indicate cell debris that is presumably from dead converted neurons (n=4 mice per group. **P* < 0.01 by Student's t-test).

Supplementary Table 1. Primary antibodies used for immunofluorescence analysis

Antibody	Host	Dilution	Source
GFP (Green fluorescent protein)	Chick	1:600	Aves Labs
GFP (Green fluorescent protein)	Rabbit	1:500	Invitrogen
Tuj-1 (Neuronal Class III β-Tubulin)	Mouse	1:10000	Covance
Tuj-1 (Neuronal Class III β-Tubulin)	Rabbit	1:10000	Covance
Map-2 (microtubule-associated protein 2)	Mouse	1:750	Sigma
NeuN (Neuronal nuclei protein)	Mouse	1:500	Chemicon
DCX (Doublecortin)	Goat	1:150	Santa Cruz Biotechnology
GABA (Gamma aminobutyric acid)	Rabbit	1:1000	Sigma
GAD-67 (Glutamic acid decarboxylase 67)	Mouse	1:100	Hybridoma Bank
vGlut1 (Vesicular glutamate transporter 1)	Mouse	1:100	UC Davis
vGlut2 (Vesicular glutamate transporter 2)	Mouse	1:100	UC Davis
Synapsin-1	Rabbit	1:100	Cell Signaling
Synaptotagmin-1	Mouse	1:200	DSHB
BrdU (5-bromo-2-deoxyuridine)	Rat	1:500	Accurate Chemical
Ki67	Rabbit	1:500	Novocastra
HuNu (Human nuclei protein)	Mouse	1:50	Millipore
NGN2	Rabbit	1:500	Chemicon
